# A Comparison of Supervised Machine Learning Algorithms and Feature Vectors for MS Lesion Segmentation Using Multimodal Structural MRI

**DOI:** 10.1371/journal.pone.0095753

**Published:** 2014-04-29

**Authors:** Elizabeth M. Sweeney, Joshua T. Vogelstein, Jennifer L. Cuzzocreo, Peter A. Calabresi, Daniel S. Reich, Ciprian M. Crainiceanu, Russell T. Shinohara

**Affiliations:** 1 Department of Biostatistics, The Johns Hopkins University, Baltimore, Maryland, United States of America; 2 Translational Neuroradiology Unit, Neuroimmunology Branch, National Institute of Neurological Disease and Stroke, National Institute of Health, Bethesda, Maryland, United States of America; 3 Department of Statistical Science, Duke University, Durham, North Carolina, United States of America; 4 Center for the Developing Brain, Child Mind Institute, New York, New York, United States of America; 5 Department of Radiology, The Johns Hopkins University School of Medicine, Baltimore, Maryland, United States of America; 6 Department of Neurology, The Johns Hopkins University School of Medicine, Baltimore, Maryland, United States of America; 7 Department of Biostatistics and Epidemiology, Center for Clinical Epidemiology and Biostatistics, Perelman School of Medicine, University of Pennsylvania, Philadelphia, Pennsylvania, United States of America; Centre Hospitalier Universitaire Vaudois Lausanne - CHUV, UNIL, Switzerland

## Abstract

Machine learning is a popular method for mining and analyzing large collections of medical data. We focus on a particular problem from medical research, supervised multiple sclerosis (MS) lesion segmentation in structural magnetic resonance imaging (MRI). We examine the extent to which the choice of machine learning or classification algorithm and feature extraction function impacts the performance of lesion segmentation methods. As quantitative measures derived from structural MRI are important clinical tools for research into the pathophysiology and natural history of MS, the development of automated lesion segmentation methods is an active research field. Yet, little is known about what drives performance of these methods. We evaluate the performance of automated MS lesion segmentation methods, which consist of a supervised classification algorithm composed with a feature extraction function. These feature extraction functions act on the observed T1-weighted (T1-w), T2-weighted (T2-w) and fluid-attenuated inversion recovery (FLAIR) MRI voxel intensities. Each MRI study has a manual lesion segmentation that we use to train and validate the supervised classification algorithms. Our main finding is that the differences in predictive performance are due more to differences in the feature vectors, rather than the machine learning or classification algorithms. Features that incorporate information from neighboring voxels in the brain were found to increase performance substantially. For lesion segmentation, we conclude that it is better to use simple, interpretable, and fast algorithms, such as logistic regression, linear discriminant analysis, and quadratic discriminant analysis, and to develop the features to improve performance.

## Introduction

Machine learning is a popular perspective for mining and analyzing large collections of medical data [1]–[3]. We focus on the extent to which the choice of machine learning or classification algorithm and the feature extraction function impact performance in one problem from medical research – supervised multiple sclerosis (MS) lesion segmentation in structural magnetic resonance imaging (MRI). The evaluation of the classification algorithms employed in supervised lesion segmentation methods is not only a function of classification accuracy. Depending on the application, computational efficiency and interpretability may be valued at the cost of classification accuracy. Therefore, our evaluation also includes the computational time and resources required by each algorithm and the interpretability of the results produced by the algorithm. Comparison of machine learning techniques has been performed in other applications[4]–[6], but not to our knowledge in multiple sclerosis lesion segmentation. Also many of the currently available comparisons do not consider computational time.

MS is a life-long chronic disease of the central nervous system that is diagnosed primarily in young adults who will have a near normal life expectancy. Because of this, the burden of the disease is great, with large economic, social and medical costs. Between 250,000 and 400,000 people in the United States have been diagnosed with MS, and the estimated annual cost of the disease is over six billion dollars. There is currently no cure for MS, but many therapies exist for treating symptoms and delaying accumulation of permanent disability (http://www.ninds.nih.gov/disorders/multiple_sclerosis/detail_multiple_sclerosis.htm). MS is characterized by demylinating lesions that are predominately located in the white matter of the brain, and MRI of the brain is sensitive to these lesions [7]. Quantitative MRI metrics, such as the number and volume of lesions, are important clinical tools for research into the pathophysiology and natural history of MS [8]. In practice, lesion burden is determined by manual or semi-automated examination and delineation of MRI, which is time consuming, costly, and prone to large inter- and intra- observer variability [9]. Therefore development of automated MS lesion segmentation methods is an active research field [8], [10]. The problem of automated MS lesion segmentation must be addressed by a method that is both sensitive and specific to white matter lesions, and which generalizes across subjects and imaging centers.

Many machine learning algorithms have been developed for automated segmentation of MS lesions in structural MRI. Over 80 papers have been published on the topic in the last 15 years, and yet no solution to this problem has emerged as superior to other methods [10]. Each lesion segmentation method in the literature is the composition of a classification algorithm and feature extraction function applied to one or many MRI modalities. As different methods use different data sets and performance metrics, the extent to which the classification algorithm, the feature extraction function, and the interplay between the classification algorithm and feature extraction function impacts the performance of these methods is unknown. To investigate this, we examine which factors improve classification performance through the composition of nine supervised classification algorithms with six feature extraction functions. We use voxel intensities form the T1-weighted (T1-w), T2-weighted (T2-w) and fluid-attenuated inversion recovery (FLAIR) MRI modalities to train and validate performance of the combinations of classifiers and feature extraction functions. We are not proposing a new lesion segmentation method. Rather than searching for the optimal method, we explore the problem and present our insights into the tools and methods of approach.

Our findings are that, for the employed feature extraction methods, the particular classification algorithm is much less important than the careful development of the features. These findings are not unique to this problem. Hand (2006) asserts that, in practice, in classification, simple classifiers typically yield performance almost as good or better than more sophisticated classifiers [11]. This is attributed to sources of uncertainty in data that are generally not considered in the classical supervised classification paradigm. Hand refers to using complex classification algorithms as “the illusion of progress”. Our findings support this characterization.

## Experimental Methods

### Ethics Statement

The Johns Hopkins Medicine IRB acknowledged the collection and analysis of data presented in this manuscript qualifies as exempt research under the Department of Health and Human Services regulations. MRI and clinical data were previously collected as part of IRB approved research studies with written consent provided by participants. The identifiable MRI and clinical information accessed by the principal investigator were recorded in such a manner that subjects cannot be identified, directly or through identifiers linked to the subjects (e.g., no codes or links were retained to allow re-identification of individuals).

### Study Population and Experimental Methods

We consider MRI studies with T1-w, T2-w and FLAIR volumes from 98 patients with MS. The 3D T1-MPRAGE images (repetition time (TR)  = 10 ms; echo time (TE)  = 6 ms; flip angle (FA) 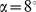; inversion time (TI)  = 835 ms, resolution  = 1.1 mm 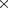
1.1 mm 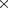
1.1 mm), 2D T2-w FLAIR images (TR  = 11000 ms; TE  = 68 ms; TI  = 2800 ms; in-plane resolution  = 0.83 mm 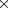 0.83 mm; slice thickness  = 2.2 mm) and T2-w volumes(TR  = 4200 ms; TE  = 80 ms; resolution  = 0.83 mm 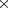
0.83 mm 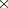
2.2 mm) were acquired on a 3 tesla MRI scanner (Philips Medical Systems, Best, The Netherlands) equipped with an 8-channel phased array head coil.

### Image Preprocessing

We preprocessed the MRI images using the tools provided in Medical Image Processing Analysis and Visualization (MIPAV) [12], TOADS-CRUISE (http://www.nitrc.org/projects/toads-cruise/), and Java Image Science Toolkit (JIST) [13] software packages. We first rigidly aligned the T1-w image of each subject into the Montreal Neurological Institute (MNI) standard space (voxel resolution 1 mm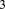). We then registered the FLAIR and T2-w images of each subject to the aligned T1-w images. We also applied the N3 inhomogeneity correction algorithm [14] to all images and removed extracerebral voxels using SPECTRE, a skull-stripping procedure [15]. A brain tissue mask is created from the extracerebral voxel removal mask by removing voxels falling below the 15th percentile of the FLAIR intensities over the mask [16]. This brain tissue mask removes cerebrospinal fluid in the ventricles and outside the brain.

## Statistical Methods

Our aim is to examine the extent to which the classification algorithm and the feature extraction function impacts the performance of lesion segmentation methods. To do so, we compare compositions of supervised classification algorithms and feature extraction functions for the classification of lesion voxels versus healthy tissue in structural MRI studies. A feature extraction function is a function that acts on the observed intensities from the MRI modalities and produces a feature vector. For each voxel of the brain, we have a “silver standard” manual lesion segmentation used to train and validate the lesion segmentation method. Manual lesion segmentations are considered a silver standard, as opposed to ground truth, as there is much inter- and intra- observer variability amongst expert segmentations. We examine lesion segmentation methods that are the composition of a classification algorithm and a feature extraction function. Here, our goal is not to find an the optimal lesion segmentation method; we instead search for insight by evaluating the performance over a set of possible classifiers and extraction functions.

### Supervised Classification Algorithms

Voxels within a brain are spatially correlated. To include spatial information, we also include functions of voxel neighborhoods as features in our voxel-level classifiers. The extent to which the residuals are still correlated after these features are included or to what extent this information can be used to further improve prediction remain open problems.

We provide a short description of the super learner, as this is not a standard classification technique employed in the neuroimaging literature. The super learner is a method for combining class estimations from different classification algorithms, by weighting the classifiers according to their prediction performance using a cross-validation loss function [17]–[20], which is referred to in the literature as ensemble learning, model stacking, or super learning. The super learner assigns each classification algorithm a coefficient weight, 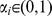, with 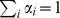. A more detailed description of the super learner and of the other supervised classification algorithms used in this analysis can be found in the Appendix S1.

Table 1 shows a summary of the classification algorithms applied to each of the feature vectors, including the R package used to apply the algorithm and, when applicable, the tuning parameter values that were searched over. We performed all modeling in the R environment (version 2.15.3, R Foundation for Statistical Computing, Vienna, Austria) with the packages AnalyzeFMRI [21], ROCR [22], MASS [23], class [23], nnet [23], mclust [24], [25], e1071 [26], randomForest [27], and SuperLearner [28].

**Table 1:** A summary of the supervised classification algorithms.

Algorithm	R package	Tuning Parameters
Logistic Regression		defaults
Linear Discriminant Analysis	MASS	defaults
Quadratic Discriminant Analysis	MASS	defaults
Gaussian Mixture Model	mclust	defaults
Support Vector Machine	e1071	cost: 1/8, 1/4, 1/2, 1, 2, 4, and 8
(with linear kernel)		
Random Forest	randomForest	number of trees = 500
		mtry = 1: dimension of the feature vector
k -Nearest Neighbor	class	k = 1,10, 100
Neural Network	nnet	size = 1, 5, 10
Super Learner	SuperLearner	all algorithms

Values for the tuning parameters for each algorithm were selected using 10-fold cross validation on the voxels in the training set and validation of the algorithms was performed on a separate set.

### Feature Extraction Functions and Vectors

**Figure 1:**
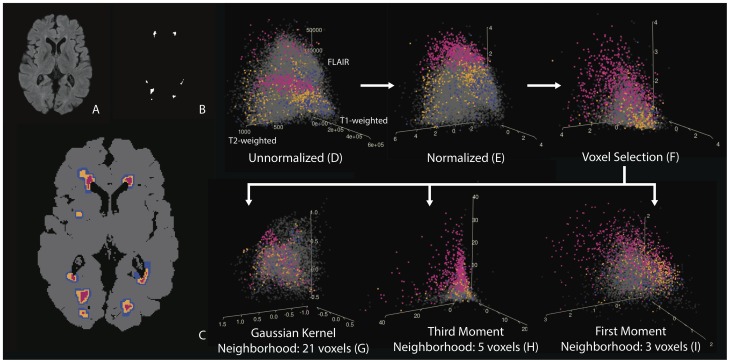
Scatter plots of the T1-w, T2-w and FLAIR voxel intensities and functions of these intensities for 10,000 randomly sampled voxels from 5 randomly sampled subject's MRI studies. Each point in the plot represents a single voxel from a study. (A–C) Color key for these plots: (A) the FLAIR volume for an axial slice from a single subject's MRI study, (B) the technician's manual segmentation for this slice and (C) the colors that are used in the plots corresponding to this slice. Lesion voxels are pink, voxels within 1 mm of a lesion voxel are orange, voxels within 2 mm of a lesion voxel are blue and all other voxels in the brain are colored grey. The arrows in the figure indicate the order that the features are created. For the unnormalized intensities there is no plane that can separate lesion voxels from non-lesion voxels, but after normalization and with the addition of features that include neighborhood information, a plane is able to separate lesion and non-lesion voxels with improved accuracy.

In this section we introduce the six feature extraction functions and the vectors these functions produce. In Figure 1, we examine 3-dimensional scatter plots of the intensities of the features that form the feature vectors. Note that while many of the feature vectors are in a higher dimension, in this figure we are only able to show 3-dimensions. Scatter plots of the T1-w, T2-w and FLAIR intensities and functions of these intensities for 10,000 randomly sampled voxels of 5 randomly sampled subject's MRI studies (a total of 50,000 voxels) are shown; each point in the plot represents a single voxel from a MRI study. We will refer to this figure throughout this section. Table 2 contains a summary of the feature vectors introduced in this section.

**Table 2:** A summary of the six feature vectors.

Feature Vector	Voxel Intensities (T1-w, T2-w, FLAIR)	Voxel Selection	Dimension
		Procedure	
Unnormalized	Observed		3
Normalized	Normalized		3
VoxelSelection	Normalized	X	3
Smoothed	Normalized	X	9
	Smoothed volumes, 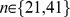		9
Moments	Normalized	X	21
	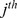 Local moment volumes, 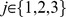 , 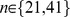 ,		
Smoothed and	Normalized	X	27
Moments	Smoothed volumes, 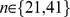		
	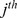 Local moment volumes, 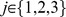 , 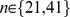		

#### Unnormalized

The unnormalized feature vector contains the observed voxel intensities (after image pre-processing) for a voxel 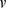 from the T1-w, T2-w and FLAIR volumes. Figure 1D shows a plot of the observed voxel intensities for the five subjects for the three volumes. Lesions appear as hyperintensities on the FLAIR and T2-w volumes and as hypointensities on the T1-w volume.

#### Normalized

**Figure 2:**
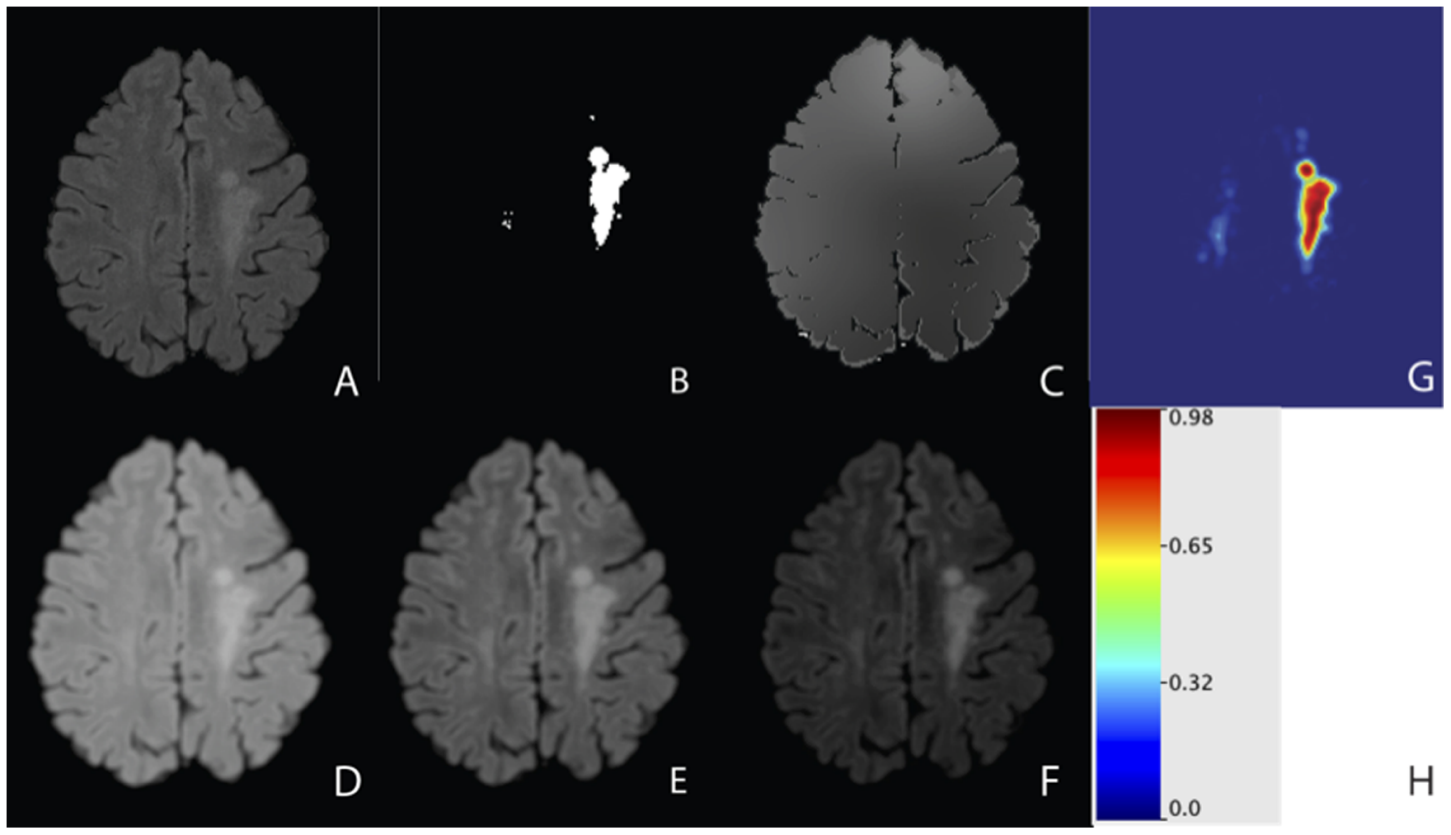
An axial slice from a single subject of the FLAIR volume with the normalization procedure, manual lesion segmentation, neighborhood functions of the FLAIR volume, and an example of a classification result. (A) FLAIR volume; (B) manual lesion segmentation; (C) FLAIR smoothed volume with a neighborhood n = 41; (D) FLAIR first local moment volume with neighborhood n = 5; (E) FLAIR second local moment volume with neighborhood n = 5; (F) FLAIR third local moment volume with neighborhood n = 5. The smoothed volumes act on large neighborhoods, while the local moment volumes act over smaller neighborhoods; (G) The probability map produced for the logistic regression classifier on the smoothed feature vector; (H) The scale of intensities in the probability map.

We normalize voxel intensities by transforming them into standard scores over the brain tissue mask [29]. The normalized feature vector contains the normalized voxel intensities from the three imaging modalities. Figure 1E shows a plot of the normalized voxel intensities from the three modalities. Figure 1A shows a slice of the FLAIR volume after the normalization procedure and Figure 2B shows the manual lesion segmentation for this slice.

#### Voxel Selection

We select candidate lesion voxels to lower computational time and restrict the modeling space. As most lesion voxels appear as hyperintensities in the FLAIR volume, we apply the brain tissue mask to the FLAIR volume and select the 85th percentile and above of voxels in the brain tissue mask as candidate voxels for lesion presence. Figure 1F shows the normalized voxel intensities for only the candidate voxels. The voxel selection procedure is not needed for simple methods but is needed for more complex algorithms.

#### Smoothed

The smoothed feature vector contains voxel intensities from the smoothed volumes, which are functions of each voxel and its neighbors. The smoothed volumes act on relatively large neighborhoods and are designed to capture anatomical information and residual intensity inhomogeneities (for a detailed discussion see [16]). Let the neighborhood of size 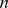 be the 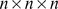 neighborhood of voxels centered at 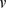. Two smoothed volumes for each imaging modality are created by 3-dimensional Gaussian smoothing of the normalized intensities for the modality over the neighborhood of size 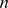 within the brain tissue mask; in this application we chose 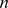  = 21 and 41. We used the FSL tool fslmaths (http://www.fmrib.ox.ac.uk/fsl) to create the smoothed volumes. Due to the high dimension of this feature vector, we cannot visualize intensities from the entire vector. Figure 1G shows the voxel intensities of the smoothed volumes for the three modalities for a neighborhood of 21 voxels for candidate voxels from the voxel selection procedure. Figure 2C shows a slice of the smoothed volume for the FLAIR, neighborhood of 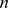 = 41.

#### Moments

In contrast to the smoothed volumes, which act on relatively large neighborhoods, the local moment volumes are designed to incorporate spatial information from nearby neighboring voxels, as a single lesion is typically comprised of multiple clustered voxels. The local moment volumes are created by taking the 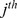 sample moment for each voxel 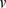 over the neighborhood of size 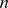 of the normalized volume over the brain tissue mask (the 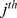 sample moment for a set of r points is defined to be 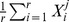). Here we use 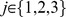 for the sample moments and 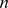 = 3 and 5 for the neighborhood size. Due to the high dimension of this feature vector, we again cannot visualize intensities from the entire vector. Figure 1H shows the voxel intensities of the third local moment volumes for the three modalities with a neighborhood of 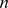 = 5 voxels for candidate voxels. Figure 1I shows the voxel intensities of the first local moment volumes for the three modalities with a neighborhood of 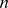 = 3 voxels for candidate voxels. Figure 2 shows an axial slice from a subject of the local moment volume for the FLAIR with a neighborhood of n = 5; Figure 2D shows the first local moment volume, Figure 2E the second, and Figure 2F the third. In the local moment volumes the contrast between lesion and other tissue is increased.

#### Smoothed and Moments

The smoothed and moments feature vector contains the intensities from the smoothed feature vector and the moments feature vector. This vector contains the normalized voxel intensities and intensities from the smoothed volumes and local moment volumes for candidate voxels from the voxel selection procedure.

### Training and Validation Studies with Manual Lesion Segmentations

For each of the 98 MRI studies we have a manual lesion segmentation made by a technician with more than 10 years of experience in delineating white matter lesions. A neuroradiologist with more than 10 years of experience in MRI of MS patients reviewed the manual segmentations and found them to be acceptable. The manual segmentations were made only for white matter lesions and were made using the FLAIR and T1-w volumes. To assess the performance of the algorithms on each of the feature vectors, we randomly assigned 49 of the MRI studies to a training set and the remaining 49 studies were used for validation. We fit the algorithms on a set of 500 voxels sampled from each of the training MRI studies (for a total of 24,500 voxels). This was done in order to reduce the time needed to fit each algorithm, as many of the algorithms with tuning parameters required a substantial amount of time to fit on larger sets of voxels. We investigate the effect on performance and computational time of this and further downsampling in the Results section. Values for the tuning parameters for each algorithm were selected using 10-fold cross validation on the voxels in the training set. We validated on the entire brain volume for each of 49 studies in the validation set. Table 3 shows a summary of the training set, the training set after the voxel selection procedure and the validation sets.

**Table 3:** A summary of the training set, training set after the voxel selection procedure has been applied, and the validation set.

	Training Set	Training Set with Voxel Selection	Validation Set
Number of Subjects	49	49	49
Number of Lesion Voxels (%)	288 (1.2%)	273 (7.5%)	458,407 (0.8%)
Total Number of Voxels	24500	3664	54,751,501

Subjects were randomly assigned to the training or validation set. All training, including tuning of algorithm parameters with 10-fold cross validation, was performed on the training set.

### Measures of Outcome and Agreement

In the Results section, we report the partial Receiver Operating Characteristic (pROC) curve and scaled partial Area Under the Curve (pAUC) as measures of algorithm accuracy. The pROC and pAUC are calculated for false positive rates of 10% and below on the validation set, using the manual lesion segmentations as a “silver standard” of truth. The scaled pAUC is computed by dividing the pAUC by the false positive rate of 10% [30] and takes values between 0 and 1, with a value of 1 corresponding to perfect classification of lesion and non-lesion voxels. We also report the Dice similarity coefficient (DSC) as a measure of agreement amongst the binary segmentations produced by each algorithm. To calculate the DSC we threshold the probability map for each algorithm at a false positive rate of 0.5% in the validation set. The DSC is a measure of the overlap of two sets [31], with a DSC of 0 corresponds to no overlap and a DSC of 1 corresponds to perfect overlap. The efficiency of the algorithms, in the form of computational time for fitting the algorithms on the training set and making predictions on the validation are reported. We also examine the effect of downsampling the training set on fit time, performance and the super learner coefficient weights.

## Results

### Classification Performance

**Figure 3:**
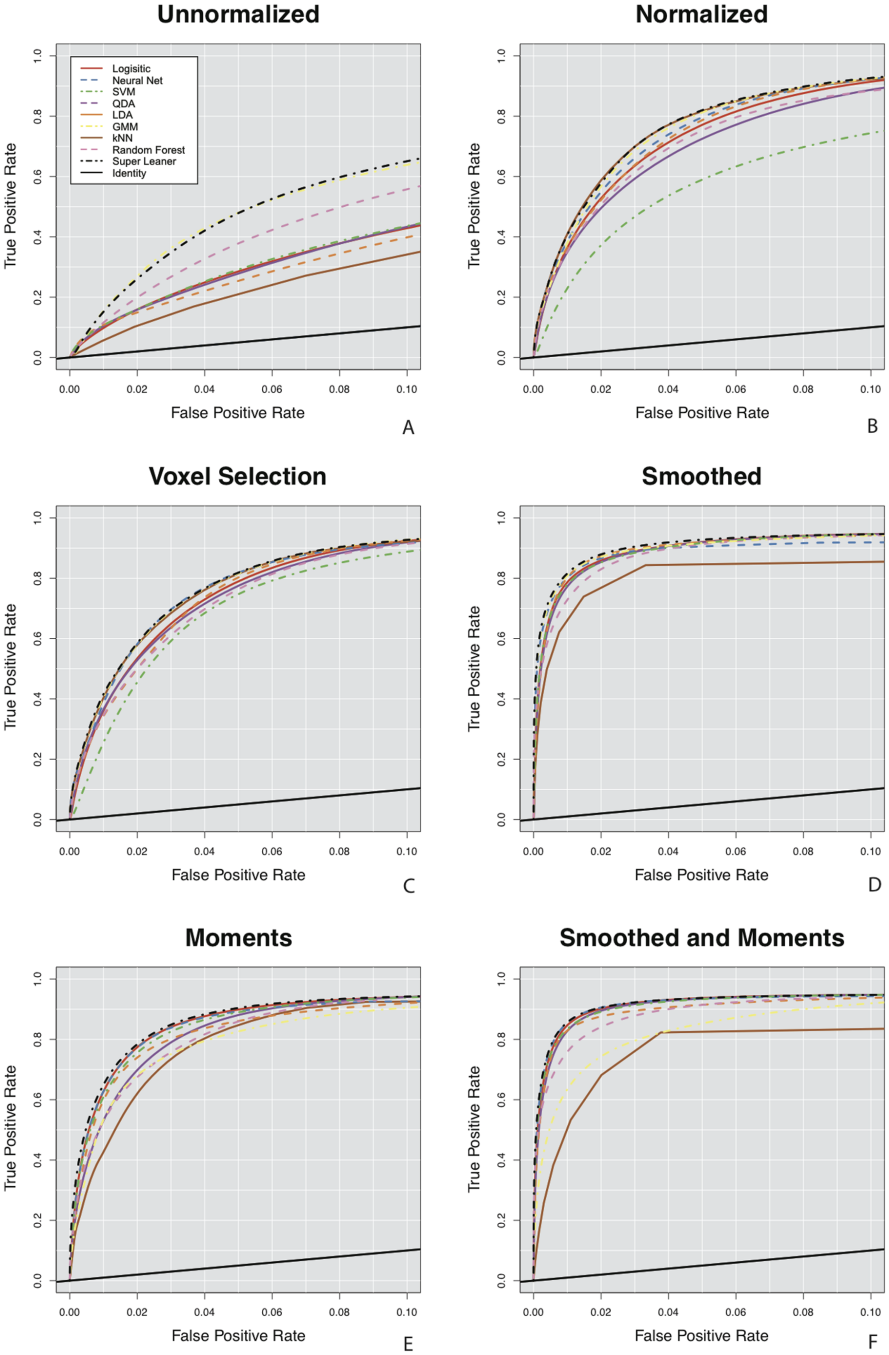
The partial Receiver Operating Characteristic (pROC) curves for the classification algorithms for false positive rates up to 10% in the validation set. The diagonal line is shown on each plot in black for reference, and represents a classifier that performs as well as chance. A plot is presented for each of the six feature vectors: (A) unnormalized, (B) normalized, (C) voxel selection, (D) smoothed, (E) moments, and (F) smoothed and moments. The performance of the simpler classification algorithms on the feature vectors with features including spatial information are superior to that of the more complex classifiers on the original features on the unnormalized feature vector.

**Figure 4:**
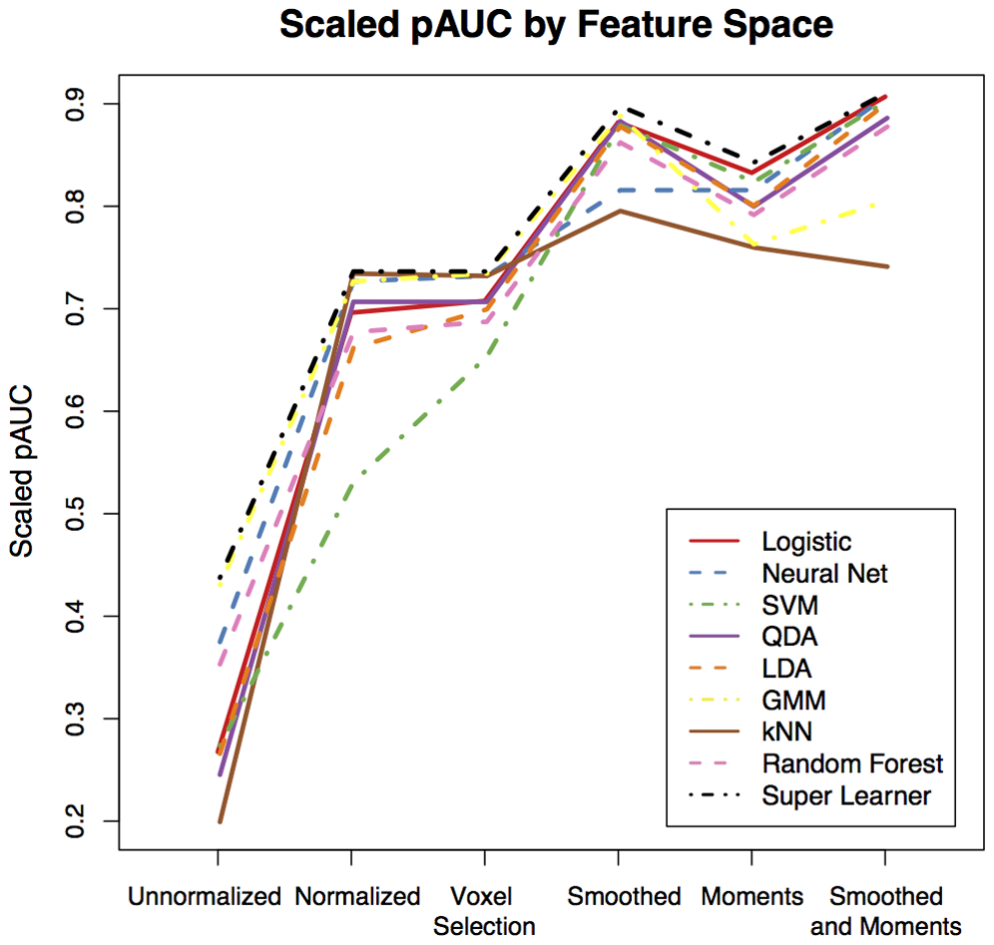
The scaled partial Area Under the Curve (pAUC) for each algorithm on each feature vector. The differences in scaled pAUC comes more from differences in feature vectors than differences in classification algorithms. The scaled pAUC of the simpler classification algorithms in the developed feature vectors are larger than that of the more complex classifiers on the original features in the unnormalized feature vector.

Figure 3 shows the pROC curves for false positive rates up to 10% for the nine classification algorithms organized by the feature vectors, with a plot for each of the six vectors: unnormalized (Figure 3A), normalized (Figure 3B), voxel selection (Figure 3C), smoothed (Figure 3D), moments, (Figure 3E) and smoothed and moments (Figure 3F). We also report scaled pAUC for false positive rates of 10% for each feature vector in Figure 4. The spread in the pROC curves in Figure 3 and the scaled pAUC in Figure 4 comes more from the different feature vectors than the classification algorithms. When fitting the algorithms on the unnormalized feature vector, algorithms perform better and worse relative to one another. In Figures 3 and Figure 4 we see that more complex algorithms, such as the super learner, Gaussian mixture model, neural network, and random forest, perform better than simpler algorithms. We define complexity as a function of an algorithm's decision boundary – for example algorithms with linear decision boundaries are relatively simpler than algorithm with nonlinear decision boundaries [32]. After normalization and the addition of features that include neighboring information, the performance of the algorithms begin to converge. All of the algorithms perform worse on the moments feature vector than the smoothed feature vector, even thought the dimension of the moments feature vector is much higher than that of the smoothed feature vector (21 versus 9). In this application and in terms of predictive performance, the smoothed volumes are better features for classifying lesions than the local moment volumes. Also, two of the algorithms, the Gaussian mixture model and the k-nearest neighbors, have a decreased performance relative to other algorithms on the validation set in the smoothed and moments feature vector.

The performance of the simpler classification algorithms on the developed feature vectors is superior to that of the more complex classifiers on the observed MRI intensities in the unnormalized feature vector. In Figure 3 we see that the best performing algorithms on the unnormalized feature vector, the super learner and Gaussian mixture model, exhibit inferior performance to all algorithms on the developed feature vectors. An intuition for this result from the data is provided in the plots of Figure 1. Figure 1D shows a plot of the intensities from the unnormalized T1-w, T2-w and FLAIR volumes. There is no plane that can separate lesion voxels from non-lesion voxels. This may be the reason as to why algorithms with nonlinear decision boundaries perform better on the unnormalized feature vector. After normalization in Figure 1E, a plane is able to separate lesion and non-lesion voxels with improved accuracy. With the addition of the smoothed and moment volumes (Figure 1G–H), classification accuracy is further improved.

Note that many of the algorithms required a range of user-supplied tuning parameters (Table 3). We invested much time in deciding which tuning parameters to allow the algorithms to search over, as using the incorrect parameters greatly diminished the performance of these algorithms. As there are an infinite number of parameters than can be searched, we can never be certain that we have decided upon the optimal parameters, and therefore we prefer using algorithms that are completely informed by the data.

### Algorithm Agreement

**Figure 5:**
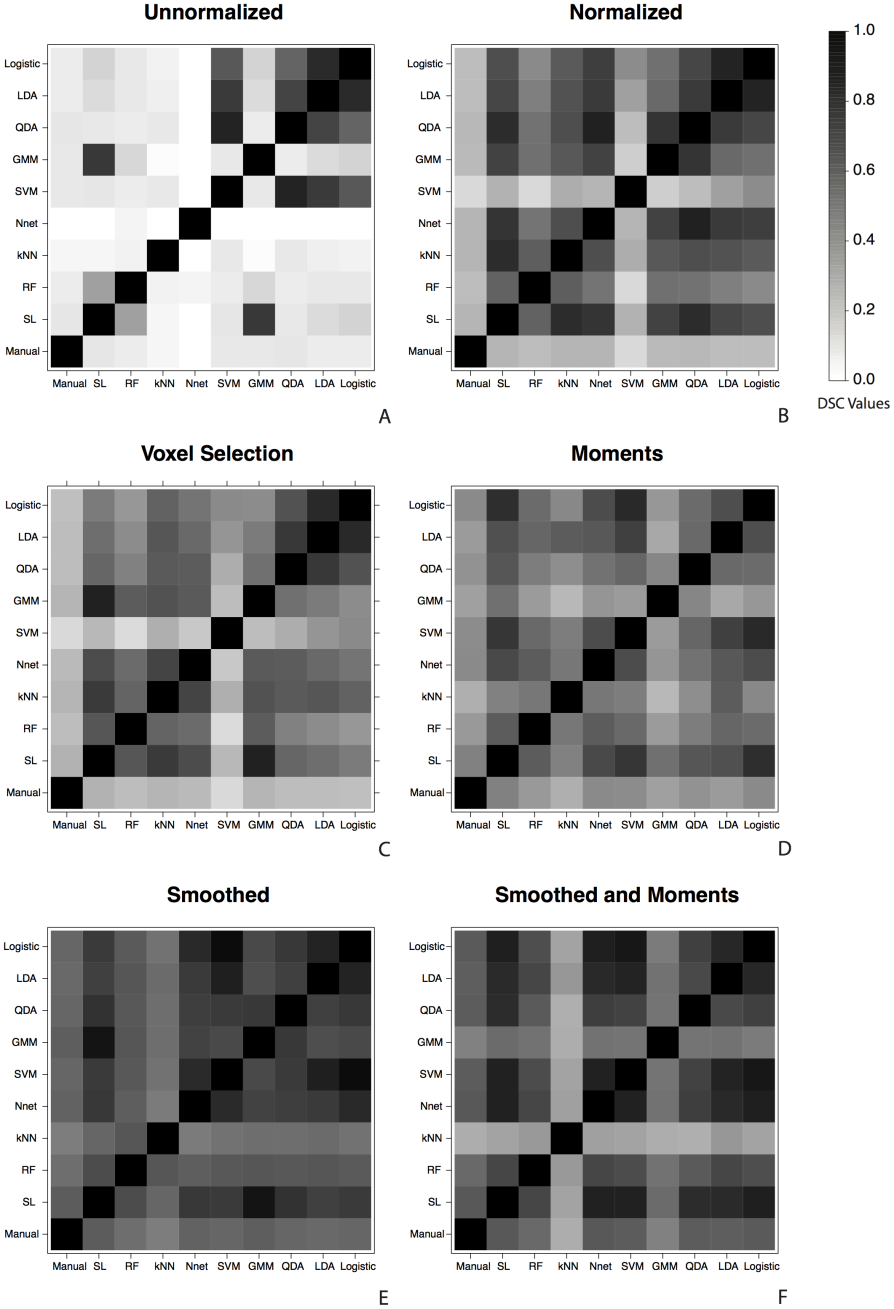
The Dice similarity coefficient (DSC) for all pairs of classification algorithm segmentations and manual segmentations. The binary segmentations for each classification algorithm are at a threshold of false positive rate  = 0.5% in the validation set. A plot is presented for each of the six feature vectors: (A) unnormalized, (B) normalized, (C) voxel selection, (D) smoothed, (E) moments, and (F) smoothed and moments. On the developed feature vectors, the class labels assigned to the voxels for each algorithm are similar. This shows that not only are the overall predictive performances of the methods similar on these vectors, but the resulting segmentations from each method are also similar.

To assess the agreement in the class label assigned to a voxel by each algorithm, we report the DSC for all pairs of binary segmentations from the classification algorithms and manual segmentations on each feature vector. We found these results to be robust to the choice of false positive rate threshold. Figure 5 shows the DSC for each of these pairs, with a plot for each of the six feature vectors: unnormalized (Figure 5A), normalized (Figure 5B), voxel selection (Figure 5C), smoothed (Figure 5D), moments, (Figure 5E) and smoothed and moments (Figure 5F). The DSC is shown as shades of gray in the plots, with darker shades indicating a DSC value closer to one. For the unnormalized feature vector, there is poor overlap between the class labels assigned by most algorithms and there is poor overlap with all algorithms and the manual segmentation. On this vector, the DSC for all pairs of the logistic regression, linear discriminant analysis, quadratic discriminant analysis and support vector machine algorithms are large. Also the super learner and Gaussian mixture model as well as the super learner and the random forest also have relatively large DSC, which is to be excepted as the super learner assigns relatively high coefficient weights to both of these algorithms on the unnormalized feature vector. On the normalized and voxel selection feature vectors, we see high DSC for all pairs of the algorithms, excluding the support vector machine, but see a low DSC with all algorithms and the manual lesion segmentation. On the smoothed, moments, and smoothed and moments feature vectors we see that the DSC for the manual lesion segmentations and all algorithms are large. Also the DSC for all pairs of the algorithms are also large (excluding the Gaussian mixture model and k-nearest neighbors algorithms on the smoothed and moments feature vector).

This plot reiterates that the performance of the simpler classification algorithms on the developed feature vectors are superior to that of the more complex classifiers on the observed MRI intensities in the unnormalized feature vector; the DSC for the class labels produced by all algorithms and the manual lesion segmentations are much larger on the moments, smoothed, and smoothed and moments feature vectors than those on the unnormalized feature vectors. The plot also shows that on the developed feature vectors, the class labels assigned to the voxels for each algorithm are similar. Not only are overall performance of the methods similar, but the segmentations produced from each method are also similar for these vectors.

### Computational Time

**Figure 6:**
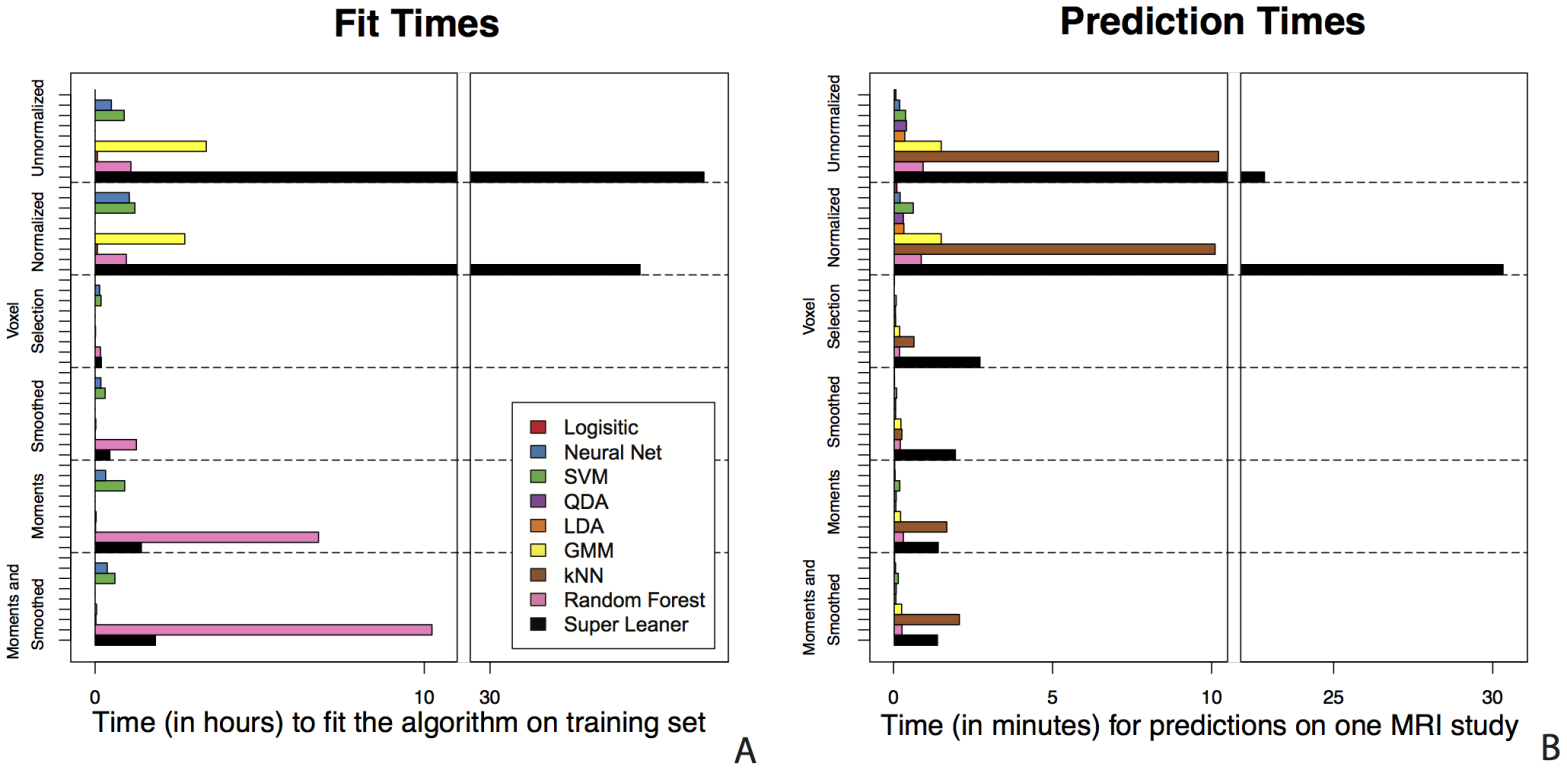
(A) The time in hours required to fit the algorithm on each feature vector and (B) the time in minutes required to make a prediction for a single MRI study from the fitted algorithms. Both of the bar plots are partitioned into the six feature vectors on the horizontal axis. The simpler algorithms without tuning parameters require significantly less computational time than more complex methods.

The bar plots in Figure 6 show the time to fit each of the classification algorithms and to make predictions on a new MRI study; Figure 6A reports the time in hours required to fit the algorithm on each feature vector and Figure 6B reports the time in minutes required to make a prediction for a single MRI study from the fitted algorithms. All of the classification algorithms were fit and made predictions on a single core to allow for accurate comparison of computing times, although many of these algorithms can be run in a parallel computing environment to decrease computation time. In Figure 6 we see that simpler algorithms without tuning parameters, such as logistic regression, linear discriminant analysis, and quadratic discriminant analysis, require significantly less computational time. These methods take under a minute to make predictions and on the feature vectors that include the smoothed and local moment volumes and these methods have comparable performance to the more complex methods. This suggests that for this application, the computational burden of the super learner is not justifiable. Note that the relative fit time for the random forest is larger than the super learner in the smoothed, moments, and smoothed and moments feature spaces. This is attributed to the mtry tuning parameter, the number of variables sampled at each split of the decision tree, which searches over a number of parameters equal to the dimension of the feature vector. The random forest takes longer to tune than the super learner in this space and this difference can be attributed to the respective packages implementation of model tuning; the SuperLearner package calls the randomForest package to fit the random forest, but selects tuning parameters internally.

Computational time to fit the algorithm on the training data and make predictions for a new MRI study is an important consideration when choosing the appropriate classification algorithm in the application of lesion segmentation. This is especially important for making predictions for a new MRI study (Figure 6B). The algorithm may only need to be trained once and, as shown in this application, and can be trained on a relatively small number of voxels to reduce computational time. In research or clinical settings, fast algorithm predictions are desired, as lesion segmentations may be required for hundreds or thousands of studies. The two algorithms with the best predictive performance on the normalized feature vector, the super learner and the k –nearest neighbors, would not scale well; the algorithms take 10 and 30 minutes respectively to make predictions for a new MRI study. Even on the feature vectors that use the voxel selection procedure, k-nearest neighbors and the super learner often take between two to three minutes to make predictions for a single MRI study, while many of the other algorithms take only a few seconds to make these predictions.

### Downsampling the Training Set: Classification Performance and Computational Time

**Figure 7:**
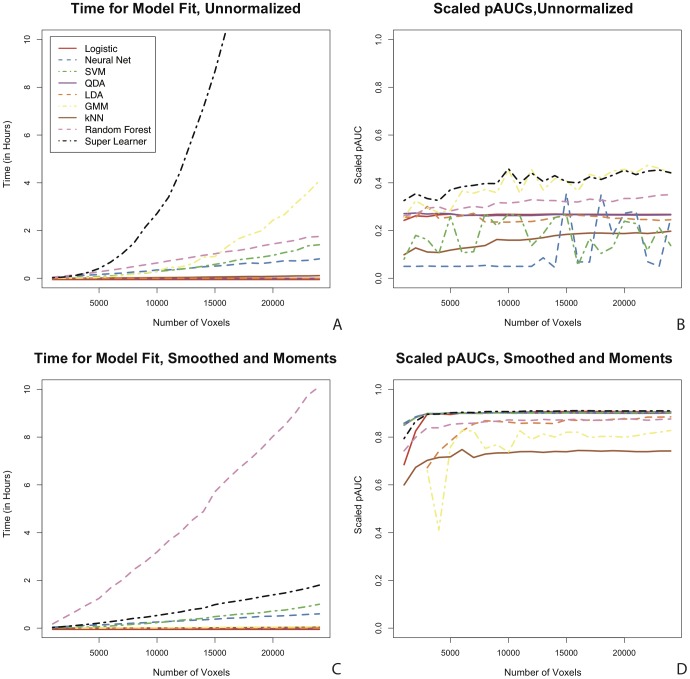
The impact of downsampling the training set on computational time and classification performance. Time in hours to fit the algorithm (left column) and scaled pAUC for false positive rates up to 10% (right column) versus the number of voxels the algorithm is fit on for the unnormalized (A,B) and smoothed and moments feature vectors (C,D). Here we see the effectiveness of downsampling the training set as the performance of the algorithms is not impacted and the computational time is significantly lowered.

In Figure 7 we investigate the impact of the number of voxels used to fit the algorithms on prediction accuracy and computation time. We examine the scaled pAUC for false positive rates up to 10% and the time to fit the algorithm on the unnormalized and smoothed and moments feature vectors. We sample 1,000 to 24,000 voxels (without replacement) by increments of 1,000 from the 24,500 voxels in the training set and fit the algorithms on each of these samples. Figure 7A shows the scaled pAUC versus the number of voxels the algorithm is fit on for the unnormalized feature vector and Figure 7B shows the time to fit the algorithm. Figure 7C and Figure 7D show the same for the smoothed and moments feature vector. At 10 hours we cut off the algorithm fit time on the plots; the super learner on the unnormalized feature vector took approximately 30 hours to fit on 24,000 voxels. The vertical axis for the smoothed and moments feature vector shows the number of voxels before the candidate voxel selection procedure is performed, as this procedure is part of the feature vector space, so the actual number of voxels the algorithm is fit on is around 15% of this size.

In Figure 7 we see the effectiveness of downsampling the training set to reduce computational time, without impacting performance. The performance of the algorithms is not impacted and the computational time is significantly lowered. On the unnormalized feature vector (Figure 7B) the performance of some algorithms varies as the number of voxels increases, especially the support vector machine and the neural network. On the smoothed and moments feature vector the performance is much more consistent. After the algorithm is fit on around 3000 voxels, the scaled pAUC for the validation set is stable as the number of voxel the algorithm is fit on increases. This shows that downsampling the training set can be an effective tool for reducing computation time without loss of performance on a well developed feature vector. In Figure 7A and C we see that in the unnormalized feature vector space the fit times for the k-nearest neighbors, quadratic discriminant analysis, linear discriminant analysis, and logistic regression appear to stay relatively constant as the number of voxels on which the algorithm is fit increases from 1000 to 24,000. The random forest, support vector machine, and neural network appear to increase linearly in computation time and the super learner and Gaussian mixture algorithm both appear to increase exponentially. The required time for the super learner, support vector machine, and neural network increase linearly and the others stay relatively constant.

### Super Learner Coefficients

**Figure 8:**
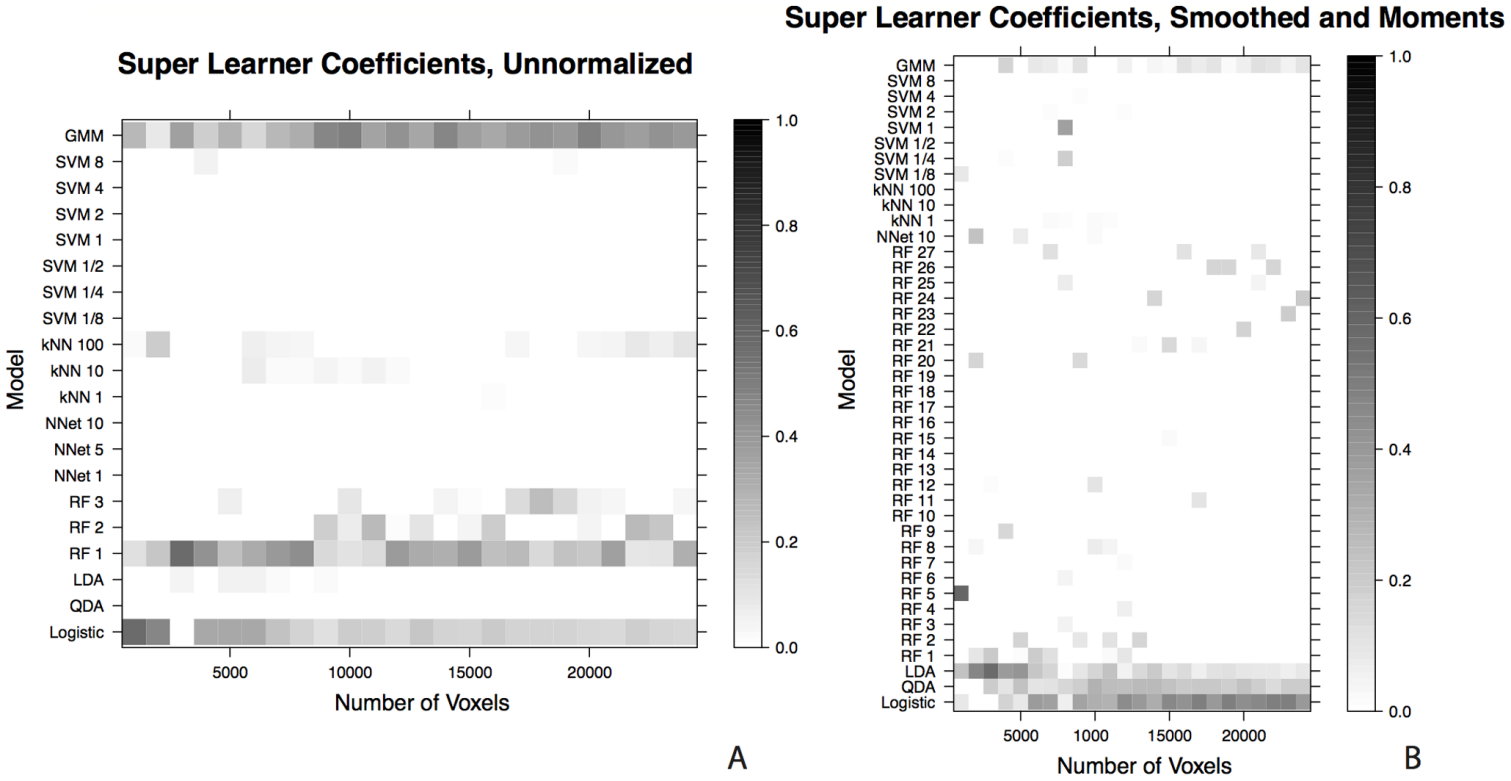
The super learner coefficient versus the number of voxels the algorithm is fit on for the (A) unnormalized and the (B) smoothed and moments feature vectors. As the number of voxels used to fit the algorithm changes, the super learner consistently assigns large weights to the same small number of algorithms. For the unnormalized feature vector, high coefficient weights are selected for the logistic regression, one of the random forest tuning parameters, and the Gaussian mixture model. On the smoothed and moments feature vector, the super learner favors the less complex algorithms: logistic regression, the quadratic discriminant analysis, and the linear discriminant analysis. Some weight is also assigned to the Gaussian mixture model and the random forest.

We examined the coefficient weights from the super learner algorithm in Figure 8. Figure 8A shows the weights, as shades of gray (darker indicating higher weight), on the unnormalized feature vector and Figure 8B shows the weights for the smoothed and moments feature vector. The weights are examined versus the number of voxels fit by the algorithm, as in Figure 7. The horizontal axis of the plots show the algorithms and tuning parameters and the vertical axis shows the number of voxels on which the algorithm was fit. The super learner is designed to select and combine the classification algorithms that perform best, by cross-validation. In Figure 8, we see that, within the same feature vector space, as the number of voxels used to fit the algorithm changes, the super learner consistently assigns large weights on the same small number of algorithms. For the unnormalized feature vector, high weights are selected for the logistic regression, one of the random forest tuning parameters, and the Gaussian mixture model. For the smoothed and moments feature vector, the super learner favors the less complex algorithms, with corresponding explicit statistical models: logistic regression, the quadratic discriminant analysis, and the linear discriminant analysis. Some weight is also assigned to the Gaussian mixture model and the random forest, although the random forest tuning parameter is unstable as the number of voxels increases.

### Interpretability

Interpretability of the algorithm is also desirable. Understanding which features improve the performance of the algorithms provides an insight into the problem of lesion segmentation and how the method may generalize; not all algorithms have the ability to provide this information. The logistic regression produces a set of coefficients with an explicit statistical interpretation, as these coefficients inform which features are most important in the context of lesion segmentation. A neural network with only one hidden layer has similar properties, but with the addition of more hidden layers, the meaning of the neural network coefficients becomes less clear. The support vector machine produces a hyperplane decision boundary for lesion and non-lesion voxels. Like a neural network with many hidden layers, the support vector machine does not provide much insight into the underlying classification problem, although recent advancements have been made to provide analytical tools for statistical inference in this framework [33]. The linear discriminant analysis produces a mean vector for each class and a shared covariance matrix for the two classes. Quadratic discriminant analysis produces a mean vector for each class and a covariance matrix for each class. The mean vector and covariance matrix or matrices from these algorithms can also be interpreted and provide information about the classification problem. The Gaussian mixture model has a similar interpretability as the linear discriminant analysis and the quadratic discriminant analysis, in that it fits a mixture of normal distributions to each class. But, in the smoothed and moments feature space, the Gaussian mixture model performs worse than many of the other algorithms. The k-nearest neighbors algorithm, random forest, and super learner all provide little intuition about the underlying classification problem, and only provide computationally complex rules for making predictions. The k-nearest neighbors algorithm also has diminished performance in the smoothed and moments feature space. While there is insight to be gained from the algorithms that the super learner assigns weights to (Figure 8), the super learner is not a practical algorithm in this context because of the computational time.

## Discussion

In this paper, we investigated the extent to which the classification algorithm, the feature extraction function, and the interplay between classification algorithm and feature extraction function impacts the performance of a lesion segmentation method. We did not search for the optimal classification method, but instead evaluated performance over a set of possible of segmentation methods, each consisting of a feature extraction function and supervised classification algorithm, to gain insight into the problem. Our findings are, for this problem, that after careful development of the feature vectors with the addition of thoughtfully designed features, the difference in classification algorithm performance disappears. The spread in the pROC curves in Figure 3, the scaled pAUC in Figure 4, and the DSC in Figure 5 are attributed to the differences in feature vectors rather than differences in classification algorithms. The performance of the simpler classification algorithms on the developed feature vectors is superior to that of the more complex classifiers on the observed MRI intensities in the unnormalized feature vector. The resulting lesion segmentation on the feature vector with additional features that incorporate spatial information are also very similar. We therefore limit our assessment of the lesion segmentation methods to those feature vectors with the best predictive performance. And as predictive performance of the majority of the algorithms on these vectors is almost indistinguishable, choosing the appropriate algorithm is a function of (1) time to fit the algorithm, (2) time to make predictions for a new MRI study, (3) interpretability of the algorithm. Using these criteria, algorithms such as logistic regression, linear discriminant analysis, and quadratic discriminant analysis and working on development of the feature vector yields the best performance.

We have shown that the development of the feature vectors greatly increases the predictive performance in this application. Much of this improvement is explained by features using intensity information over the entire brain, not just the information at the voxel-level. The normalization procedure transforms intensities into standard scores of the brain tissue mask, using the sample mean and standard deviation across the mask. The smoothed volumes and local moment volumes also use additional information from neighboring voxels. In the feature vectors containing intensities from the smoothed volumes and local moment volumes, the dimension of the feature vector is increased. The addition of features can often improve the performance of a classifier, especially when very few original features are available[34]. All of the algorithms perform better on the smoothed, moments and smoothed and moments feature vectors, than they do on the feature vectors with lower dimension. It is also useful to note, that in this application, the smoothed volumes which use a much larger neighborhood are a better features for classifying lesions than the local moment volumes, which use information in a smaller neighborhood. While one might expect performance to be higher on the moment volumes than the smoothed volumes, as the dimension is higher in the space of the moment volumes, it has been shown that the addition of noisy features can diminish classification performance.[34] A number of image smoothers can be used to incorporate spatial information. We chose the smoothed volumes and local moment volumes to illustrate a smoother over a relatively large neighborhood and relatively small neighborhood, respectively. We refer the reader to the literature on image smoothing in both computer vision and neuroimaging for a more complete discussion of smoothers, a review of which can be found in “The Image Processing Handbook” [35].

We performed all modeling in the R environment, using the standard implementations of each of the classification algorithms. If a classification algorithm with a high computational time exhibited an improvement in classification accuracy over other algorithms, this algorithm could be tailored to reduce computational time. There are many techniques to reduce computational time, such as more computationally efficient training and testing implementations [36]–[38], the use of parallel computation or graphics processing units [39], and programming in other languages such as Python[40]. In the application of lesion segmentation, we did not observe an improvement in classification accuracy that would merit improving computation efficiency.

Another concern is the behavior of the classifiers on unbalanced data. Many of the classification algorithms employed in the analysis have been shown to perform poorly on unbalanced training data, such as k-nearest neighbors, neural networks, and support vector machines [41], [42]. In Table 3 we show the distribution of the lesion and non-lesion voxels in the training set. For feature extraction functions without the voxel selection procedure, 1.2% of the voxels contain lesions. For feature extraction function with voxel selection, 7.5% of the voxels contain lesion. While voxel selection was performed to lower computation time, it can also be thought of as a method of balancing the training data set. It is similar to the method of down-sizing [42], where random elements of the over-sized class are randomly eliminated from the training set. One difference is that in the voxel selection procedure, voxels were not removed at random, but were removed using a threshold on the FLAIR volumes. Other methods for balancing the training set could also be applied to lesion segmentation.

There is a large literature on supervised machine learning algorithms for segmentation of MS lesions in structural MRI. From this literature it is difficult to determine the extent to which the classification algorithm and the feature extraction function impacts the performance of the lesion segmentation algorithms. Each of the methods reports different performance metrics on different datasets. The majority of supervised machine learning algorithms in the literature are a composition of a single classification algorithm and feature extraction function. Most of the feature vectors from lesion segmentation methods in the literature contain intensity normalized voxel intensities, the most popular of which is histogram matching [43]. Other common features are functions of neighborhoods of an image voxel [16], [44]–[49] or location information from an anatomical atlas [44], [47], [50]–[52]. The supervised classification algorithm used by these methods include neural networks [45], [53]–[56], k–nearest neighbors [44], [57], bayesian classifiers [51], [58], principal component analysis classification [47], Parzen window classifiers [59], model stacking [48], [49], classification based on Markov Random Fields [46], [60], supervised learning of optimal spectral gradients [61], and random forests [50]. Kamber (1995) does compare four different classification methods: a minimum distance classifier, a Bayesian classifier, an unpruned decision tree, and a pruned decision tree and found that the Bayesian classifier performed best. [52] The only features used in the method proposed by Kamber are the MRI voxel intensities and atlas derived prior probabilities of a voxel containing lesion; the method uses neither intensity normalization nor functions of the image intensities. Many of the aforementioned lesion segmentation methods use different image pre-processing steps. In this work we evaluate the impact of classification algorithms and feature extraction functions on classification performance. Our data is resliced to 1 mm isotropic resolution, a common resolution for many image processing algorithms, even for data acquired at a lower nominal resolution. As pre-processing steps may also influence classification results, future work is needed to investigate this impact.

We investigate the choice of supervised classification algorithm and feature extraction function on the performance of lesion segmentation methods. Our findings are that the particular classification algorithm is less important than the careful development of the feature vectors. For the employed feature extraction methods, classification algorithms with a linear decision boundary (logistic regression and linear discriminant analysis) performed equally well as classifiers with nonlinear decision boundaries. Also, the performance of the simpler classification algorithms with the feature vectors containing additional features is superior to that of the more complex classifiers on the original features.

## Supporting Information

Appendix S1 **Classification Algorithms.** A description of the supervised classification algorithms used in this analysis.
(PDF)
