# Supplementary material for: A Comparison of Supervised Machine Learning Algorithms and Feature Vectors for MS Lesion Segmentation Using Multimodal Structural MRI
Source: PLoS One. 2014 Apr 29;9(4):e95753. doi: 10.1371/journal.pone.0095753 (PMC4004572; doi:10.1371/journal.pone.0095753)
Supplement: Appendix S1 — Classification Algorithms. A description of the supervised classification algorithms used in this analysis. (PDF) [file pone.0095753.s001.pdf]

Appendix S1

Classification Algorithms

Voxel-level lesion segmentation is a binary classification problem with two classes, voxels either contain lesion or do not contain lesion. We use the notation $y_v \in \{0,1\}$ to be a class label indicating whether or not voxel v of a brain image from MRI study contains a lesion. Here we provide a brief summary of each classification algorithm used in our analysis and the tuning parameters associated with the algorithm. A complete overview of these algorithms can be found in The Elements of Statistical Learning: Data Mining, Inference and Prediction [?]. For each classification algorithm, Table **??** in the Methods section shows the R package used to fit the algorithm and the values for the tuning parameters. In fitting each of the algorithms on the training set, 10-fold cross validation was used to optimize all tuning parameters.

Logistic Regression

Logistic regression is a special case of the generalized linear model, with a logistic link function. The generalized linear model with logistic link function, t, is $t\{P(y_v = 1|X = x)\} = \beta_0 + \beta^T x$, where $t(s) = \log(\frac{s}{1-s})$. The resulting decision boundary for logistic regression is linear.

Linear Discriminant Analysis

Linear discriminant analysis models each class as a multivariate Gaussian with probability distribution functions $f_0(v)$ and $f_1(v)$ with means μ_0 and μ_1 respectively. Linear discriminant analysis makes the assumption that both classes have a common covariance matrix Σ. Prior probabilities of belonging to a class are calculated from the training data and are denoted as π_0 and π_1. Then, the probability of voxel being part of a lesion is defined as $P(y_v = 1|X = x) = \frac{f_1(x)\pi_1}{f_0(x)\pi_0}$. The resulting decision boundary is linear.

Quadratic Discriminant Analysis

Quadratic discriminant analysis is similar to linear discriminant analysis, but allows for the two classes to have different covariance matrices, Σ_0 and Σ_1. The resulting decision boundary is a quadratic surface.

Gaussian Mixture Model

In a Gaussian mixture model, each class k is modeled as a mixture of normal distributions with density $P(X = x|y_v = k) = \sum_{r=1}^{R_k} \psi_{kr}\phi(X; \mu_{kr}, \Sigma), k = 0, 1$. The mixing proportions for each class, ψ_{kr}, must sum to one. Then, $P(y_v = 1|X = x) = \frac{P(X=x|y_v=1)\pi_1}{P(X=x|y_v=1)\pi_1 + P(X=x|y_v=0)\pi_0}$, where π_0 and π_1 are the prior probabilities of belonging to each class, calculated from the training data. The decision boundary for the Gaussian mixture model is multi-modal

Support Vector Machine with Linear Kernel

In a support vector machine with linear kernel, we aim to find the hyperplane that separates the two classes with the largest margin. The hyperplane that separates the two class is defined as, $\{x : f(x) = \beta_0 + \beta^t x\}$. A cost C is assigned to voxels v that are classified incorrectly, and $\sum \psi_i \leq C$, where ψ_i is the amount which the voxel is on the incorrect side of the margin. We search over the $C =$ 1/8, 1/4, 1/2, 1, 2, 4, and 8. The decision boundary for the support vector machine is linear in the implicit kernel space, but nonlinear in the original space.

Random Forest

A random forest combines multiple classification trees and these classification trees vote on which class a new voxel should be classified as. In the implementation of the random forest, we use 500 classification trees. The parameter we tune the algorithm over is the mtry parameter, the number of variables sampled at each split of the decision tree. We search over mtry = 1 to the dimension of the feature space. The decision boundary for each tree is piecewise linear.

k-Nearest Neighbors

In k-nearest neighbors to classify a new voxel, v, with features $X = x$, we calculate the distance of its features from the features of the voxels in the labeled training set, using Euclidean distance. The k voxels in the training set with the smallest distance to the new voxel vote on the class of the new voxel. The tuning parameter for the k-nearest neighbor algorithm is the number of voxels to be used. We search over k =1, 10, and 100 neighbors.

Neural Network

In a single hidden layer neural network, derived features, Z, are created from linear combinations of the input features, $X : Z_p = \sigma(\alpha_{op} + \alpha_p^T X), p \in \{1, ..., P\}$. These derived features Z are called hidden units. We elected to use a sigmoid activation function σ, $\sigma(s) = \frac{1}{(1+\exp(-s))}$, as this is typical. The outcome, y_v, is then modeled as a function of the hidden units: $y_v = t(\beta_0 + \beta^T z)$. We use a single hidden layer neural network with sigmoid activation function; 10-fold cross validation is used to select the number of hidden units in the algorithm and search over P = 1, 5, and 10.

Super Learner

The super learner is a method for combining class estimations from different classification algorithms,by weighting the classifiers according to their prediction performance using a cross-validation loss function. We use the super leaner algorithm with 10-fold cross validation with mean squared error loss, but any other cross validation or loss function may be used. The super learner requires a library of K supervised classification algorithms. To train the super learner with 10-fold cross validation, the training set, T, of size n voxels is partitioned into 10 equal samples. For each of these 10 samples, the K algorithms are trained on the remaining data in the training set. A prediction for each voxel v in the reserved sample is then made for the kth classification algorithm, h_k, denoted as $\varphi_k(v), k \in \{1, \ldots, K\}$. After this is performed for all 10 samples, a coefficient α_k for each classification algorithm is selected, to minimize the mean square error: $\frac{1}{n}\sum_{v \in T}[y_v - \sum_k \alpha_k \varphi_k(v)]^2$, under the constraint $\sum_k \alpha_k = 1$. The fitted classifiers with the coefficients α_k can then used to make predictions for new samples. We use the SuperLearner R package based on all of the other supervised classification algorithms and tuning parameters used in this analysis. To decrease prediction time for new data, algorithms with α_k coefficients close to zero are dropped during the prediction phase (the onlySL parameter is set to TRUE when making predictions; the default for this parameter is FALSE) .
